# Reply to Letter to Editor: Abdominal CT: a radiologist-driven adjustment of the dose of iodinated contrast agent approaches a calculation per lean body weight

**DOI:** 10.1186/s41747-020-00179-w

**Published:** 2020-09-22

**Authors:** Moreno Zanardo, Giovanni Di Leo

**Affiliations:** 1 Department of Biomedical Sciences for Health, Università degli Studi di Milano, Via Mangiagalli 31, 20133 Milan, Italy; 2 Radiology Unit, IRCCS Policlinico San Donato, Via Morandi 30, 20097, San Donato Milanese, Italy

**Keywords:** Abdomen, Body composition, Body weight, Contrast media, Tomography (x-ray computed)

Dear Editor,

We read with interest the comment made by Dr. Peet [1] on our article “Abdominal CT: a radiologist-driven adjustment of the dose of iodinated contrast agent approaches a calculation per lean body weight” [2]. The author states that our results do not agree with theirs and others’ experience. In their experience, they did not find a reduced among-patient variability of the liver contrast enhancement (CE) on abdominal CT when using the lean body weight (LBW) instead of the total body weight (TBW) to calculate the contrast agent volume to be injected. While we understand the author’s argument, it is important to highlight that, in our article, we do not make that direct conclusion. We only found that the variability of the liver CE decreased from underweight to obese patients; however, we did not dose the contrast agent according to the LBW. Our main finding was that, in the daily practice of our institution, radiologists do apply a subjective non-standard compensation for obese patients, by administering a contrast agent volume that is lower than theoretically calculated according to the TBW. In the Discussion, we merely speculate on the fact that radiologists make an adjustment of the contrast agent dose according to a putative LBW.

Furthermore, the hypothesis of a reduction of the liver CE variability associated with the use of LBW instead of TBW has been the rationale of a randomized controlled trial we have conducted at our institution. From preliminary analysis, data seem in line with previous studies, showing no reduction of liver CE variability when using LBW. However, we did not deem it necessary to normalize the liver CE to iodine volume, as we compare the liver CE in two randomized groups that were administered the same contrast agent and concentration.

Importantly, in our opinion, when describing parenchymal enhancement, especially of the liver, a multiparametric approach is most likely necessary. It should be a non-linear function of several variables, some of which have probably not been specifically investigated yet. Thus, a single-parameter approach, though easy to use, is inadequate. Studies are needed to develop an accurate model for prediction of the liver CE and for personalizing the contrast agent dose to be administered.

We also understand the author’s concern about the lack of a formal image quality evaluation. We are convinced that a binary diagnostic/non-diagnostic assessment could be considered too simplistic, even though directly derived from the clinical practice in a retrospective design.
